# COVID-19 Impact in the Italian Reception System for Migrants during the Nationwide Lockdown: A National Observational Study

**DOI:** 10.3390/ijerph182312380

**Published:** 2021-11-25

**Authors:** Leuconoe Grazia Sisti, Anteo Di Napoli, Alessio Petrelli, Alessandra Rossi, Alessandra Diodati, Martina Menghini, Concetta Mirisola, Gianfranco Costanzo

**Affiliations:** 1 National Institute for Health, Migration and Poverty (INMP), Health Directorate, Via di San Gallicano, 25/a, 00153 Roma, Italy; gianfranco.costanzo@inmp.it; 2 National Institute for Health, Migration and Poverty (INMP), Epidemiology Unit, Via di San Gallicano, 25/a, 00153 Roma, Italy; anteo.dinapoli@inmp.it (A.D.N.); alessio.petrelli@inmp.it (A.P.); alessandra.rossi@inmp.it (A.R.); 3 National Institute for Health, Migration and Poverty (INMP), Global Health and Health Cooperation Unit, Via di San Gallicano, 25/a, 00153 Roma, Italy; alessandra.diodati@inmp.it; 4 National Institute for Health, Migration and Poverty (INMP), Information and Statistical System Unit, Via di San Gallicano, 25/a, 00153 Roma, Italy; martina.menghini@inmp.it; 5 National Institute for Health, Migration and Poverty (INMP), Directorate General, Via di San Gallicano, 25/a, 00153 Roma, Italy; concetta.mirisola@inmp.it

**Keywords:** COVID-19, public health, migrant health

## Abstract

From the beginning of the COVID-19 pandemic, attention was raised to protect vulnerable populations, including migrants and refugees (M&R), with the claim to leave no one behind in the pandemic response. In particular, concern was expressed in M&R’s reception centres since several COVID-19 outbreaks had been observed in Europe. Our study aimed to evaluate the impact of COVID-19 in the Italian reception system in the first pandemic wave in terms of incidence and health outcomes. A national survey focusing on the lockdown period of early 2020 was performed among reception centre managers. The survey achieved reaching around 70% of reception facilities and hosts. A national cumulative incidence of 400 positive cases per 100,000 and a north–south geographical gradient were observed. Sixty-eight facilities out of the 5038 participating in the survey reported confirmed cases and few COVID-19 clusters were detected especially in accommodations with the highest facility saturation index. Positive migrants were hospitalised in 25.9% of cases and no COVID-19 related deaths were observed. The study highlighted a cumulative incidence of cases and a geographical distribution similar to that of the general resident population, showing a global COVID-19 resilience in the Italian reception system in the period of observation, well beyond the expectations.

## 1. Introduction

With more than 231 million cases worldwide and over 4,7 million [1] global deaths, the new coronavirus pandemic has disrupted and impacted health, social, and economic systems around the world. Currently, the global expansion of vaccine coverage, with more than 6 billion doses administered [1], bodes well for pandemic mitigation, although more efforts should be made to ensure that vulnerable groups and populations are not left behind (e.g., in low-income countries where only 2.2% of people have currently received at least one dose of vaccine [2]).

Since the beginning of the pandemic, the scientific community has warned about the differential impact of COVID-19 in diverse populations, in terms of clinical and socioeconomic outcomes. Scientists, as well as international bodies and organisations, have drawn attention to the need to protect and include migrants in national responses to the pandemic [3,4,5].

In this regard, both the Lancet Migration and the WHO Regional Office for Europe have highlighted that both refugees and migrants are potentially at increased risk of contracting diseases, including COVID-19, due to difficult living conditions (e.g., overcrowding and homelessness) and limited access to basic sanitation and hygiene services. This is substantially due to administrative, financial, legal, and language barriers that hinder their ability to follow public health measures (e.g., social distancing, proper hand hygiene, and self-isolation) and receive adequate care [6,7].

Especially in the first phase of the pandemic, particular concern was expressed about the reception system for migrants, refugees, and asylum seekers, highlighting overcrowding [8,9] and unavailability of personal protective equipment as key factors for the spread of SARS-CoV-2 in refugee camps and reception facilities for newly arrived migrants worldwide. At that time, outbreaks of COVID-19 were reported in refugee camps and centres in Greece, Malta, Germany, the Netherlands [10] and Finland [11]. Nevertheless, epidemiological studies analysing the impact of COVID-19 on migrants hosted in reception systems are still lacking in the literature.

Italy, which was heavily affected by the first wave of the pandemic, represents one of the main transit and destination countries for international migration flows and one of the most important entry points into Europe for people crossing the Mediterranean route. The country has seen a significant increase in the stock of international migrants since 1990 (at 5,039,637 in 2020, 8.5% of the resident population in Italy) [12]. In 2020, the number of refugees and asylum seekers being hosted was estimated to be approximately 128,000 and more than 53,000, respectively [13].

To protect migrants and refugees, Italy has endowed itself with a highly structured and multi-level reception system for migrants, who are hosted in different facilities according to their legal status and the length of stay in Italy. On their arrival in Italy, migrants first receive first aid and undergo identification procedures according to the “Hotspot Approach” presented by the European Commission in 2015 [14]. These procedures are generally performed in first reception facilities (hotspot), usually set (but not necessarily) in the proximity of a landing place where new arrivals undergo medical examinations and pre-identification procedures. After being informed about their current condition of irregular stay immigrants and about the possibility to apply for international protection, fingerprints are registered. On this occasion, special needs are detected, and new arrivals receive information on the procedures of international protection, relocation programme, and assisted voluntary return [15].

If migrants apply for international protection, they are channelled into asylum procedures and, until the international protection application is defined, asylum seekers are hosted in first-level reception centres, such as the following: first reception centres (CPA), reception centres for asylum seekers (CARA), and extraordinary reception centres (CAS), the latter to be used in the case of exceptional influx of migrants and saturation of other centres. Migrants who do not apply for international protection or do not obtain a stay permit may be sent to repatriation centres, known as CPR [16]. After obtaining the title to stay and until they are integrated into the country, refugees and beneficiaries of other protection titles enter the second level of reception, SIPROIMI “Protection System for Beneficiaries of International Protection and for Unaccompanied Foreign Minors”, that is oriented to the integration of migrants. SIPROIMI is mainly represented by small facilities and apartments widespread throughout the territory; unaccompanied minor migrants are hosted in dedicated facilities of the system (known as SIPROIMI MSNA).

Recently the SIPROIMI system was replaced by the SAI (System of accommodation and integration) that also includes the asylum seekers in its eligible population. At the time of the survey the SIPROIMI was still in place and so it is referred to in the present paper. The reception system, as a whole, is under the responsibility of the Department for civil liberties and immigration of the Ministry of Interior. Migrants, even those who do not have a legal entitlement to stay, have the right to access essential and preventive services (e.g., vaccination), as well as continuity and emergency healthcare. 

On 15 March 2020, soon after the start of the nationwide lockdown measures that Italy put into force as the first country in the world, the Italian reception system for migrants was hosting 85,324 migrants [17]. The Italian National Institute for Health, Migration, and Poverty (INMP) and the Ministry of Interior agreed to evaluate the impact of the COVID-19 pandemic in the Italian reception system for migrants in the first pandemic phase (lockdown), by investigating both the incidence and the outcomes of the SARS-CoV-2 infection among hosts as well as the implementation of COVID-19 public health measures in reception facilities if finding cases of COVID-19. The results of the survey would have to be used to guide operational decisions in pandemic control if the reception system showed particular criticality.

## 2. Materials and Methods

### 2.1. Study Design, Setting, Population, and Timing

Managers of first and second reception facilities of the Italian reception system were asked to participate in a national survey regarding the nationwide lockdown period (133 days from February 1 to 12 June 2020). The survey was launched on 11 May 2020, on an online platform managed by the INMP and remained active until 12 June 2020. The populations of interest were first and second level reception facilities (6837) active in Italy on 11 May 2020 and their guests (*n* = 85,730 migrants) (data communicated by the Italian Ministry of Interior). No significant changes in the composition of the cohort were observed during the considered period due to the lockdown measures in force. 

### 2.2. Data Collection

Reception centres managers were asked to fill out an ad hoc online questionnaire designed by INMP experts. The questionnaire was composed of two parts (A and B). Part A, “information on facilities” aimed at collecting information on facilities and guests (e.g., capacity of facilities, type of accommodation, number and nationality of guests, etc.); part B, “suspected or confirmed SARS CoV-2 positive cases,” collected information related to suspected and confirmed cases, such as comorbidity, hospitalisation, and public health measures taken by reception centres and local health authorities in cases of positivity or close contacts.

The definition of suspected and confirmed cases of SARS-CoV-2 was based on that provided by the Italian Ministry of Health [18], according to the WHO definition. In detail, the suspected case was identified as a host who (a) experienced sudden onset of at least one of the signs and symptoms, such as fever ≥ 37.5 °C, cough, and respiratory distress, without another cause fully explaining the clinical presentation or who (b) in the 14 days preceding the onset of respiratory symptoms was in close contact with a probable or confirmed case of COVID-19. A confirmed case was defined as a suspected case that tested positive on molecular testing for SARS-CoV-2. 

### 2.3. Data Analysis and Statistical Analysis

The following variables and measures were considered for the analysis: the distribution of characteristics of facilities and guests, including the facility saturation index; the proportion of suspected and confirmed cases to total guests; the geographic distribution of centres with suspected and confirmed cases; the positivity rate of suspected cases; the proportion of hospitalised cases to confirmed cases; the cumulative incidence of confirmed cases; and the management of confirmed cases. An analysis was performed for each measure, stratifying the data by the 19 Italian Regions and the 2 Autonomous Provinces.

The facility saturation index (*FSI*), used as a proxy of the crowding of facilities, was calculated as number of guests/maximum capacity of the reception centre.

(1) $$FSI=\frac{n\cdot guests}{\;maximum\;capacity\;of\;reception\;center}*100$$

Cumulative incidence and 95% confidence interval were estimated, using as population at risk the total number of guests in the facilities at the beginning of the observation period. Absolute and relative frequencies were used to describe qualitative variables. Chi-square and Fisher’s exact test were used to compare the geographical distribution of reception centres participating in the survey out of those operating in the territory, the distribution of confirmed cases by type of facility and the hospitalisation rate by presence/absence of comorbidities.

The Kruskall–Wallis test was used to investigate the difference of *FSI* among geographical macro-areas. The Mann–Whitney test was used to evaluate the difference in *FSI* between facilities with and without confirmed cases. The significance level was set at 0.05. Data were analysed using SAS^®^ 9.4 software (SAS Institute Inc., Cary, NC, USA). 

## 3. Results

### 3.1. Reception Centres and Hosts 

A total of 5,038 (73.7%) facilities out of the 6,837 registered by the Ministry of Interior during the study period participated in the survey, with regional coverage ranging from 49.8% in the Friuli Venezia Giulia Region to 88.4% in the Marche Region (see Table 1). The geographical distribution showed a significantly higher participation of reception centres in Northern Italy (75.4%) compared to the Centre (72.5%) and the South and Islands (70.1%) (*p*-value < 0.001), with a strong presence of Emilia-Romagna, Lombardy, Piedmont, and Lazio regions, which cumulatively represent 52.9% of all participating facilities (48.6% of all facilities) (Table 1).

Reception centres were primarily represented by CAS (74.5%), followed by SIPROIMI, SIPROIMI MSNA (25.1%) and others (Table 2).

The centres resulted in hosting an average of 11.8 guests per centre and offered shared rooms in 68.5% as well as mixed housing (single and shared rooms) in 21.5% of cases. 

The facility saturation index, which overall reported an average value of 79%, was significantly lower in the South (including Islands) than in the Centre and North, which reported the highest saturation index (*p*-value < 0.001) (Table 2). 

Overall, 98.2% (4947 of 5038) of participating facilities reported having sufficient personal protective equipment (PPE) (not shown in table).

The survey included 70% of refugees and migrants hosted in the Italian reception system (59,648 of the 85,730 guests reported by the Ministry of Interior as of 31 May 2020) (Table 3). The majority of refugees and migrants were hosted in Northern Italy (51.1%), compared to 19.8% in Central Italy and 29.1% in the Southern Italy and Islands. The migrants hosted came primarily from Western Africa and Southern Asia, with Nigerians covering more than a quarter of the total.

### 3.2. Suspected and Confirmed COVID-19 Cases 

#### 3.2.1. Geographical Distribution and Characteristics of Facilities

The number of suspected and confirmed cases was 572 (1% of the total number of guests) and 239 (0.4%), respectively. Suspected cases were found in 169 facilities, and 90.2% of suspected cases were recorded in facilities located in the North of Italy, 7.2% in the Centre and 2.6% in the South and Islands. 

A positivity rate of 41.8% of suspected cases was recorded.

**Figure 1:**
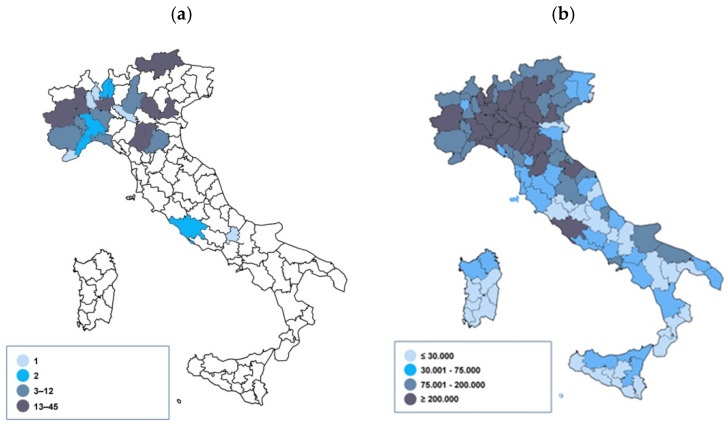
Geographical distribution of confirmed cases by province (quartiles) in the reception system (**a**) and in Italy * (**b**). Time period: For reception system: 1 February–12 June 2020, for Italy: 24 February–12 June 2020. * Source: Italian Civil Protection Department.

Confirmed cases were detected in 68 facilities participating in the survey, almost all located in Northern Italy (66; 97.1%, of which 19 in the Lombardy Region and 15 in Piedmont), 1 in the Centre (Lazio) and 1 in the South and Islands (Molise). In particular, the highest number of cases was recorded in the provinces of Milan, Bolzano, and Turin. Figure 1 shows the geographical distribution (by province) of cases confirmed in the reception system compared with that of the resident population in Italy during the same period.

Thirty-two out of 68 (47.1%) facilities reported one confirmed case, 15 (22.1%) two cases, and 21 (30.9%) three or more cases. Specifically, confirmed cases in facilities ranged from 1 to 36 cases (mean 3.51, standard deviation 5.72; median 2, interquartile range 2).

The cumulative incidence of confirmed cases in the receiving system and in the Italian resident population is shown in Table 4.

The overall cumulative incidence of confirmed cases was similar between the receiving system and the Italian resident population (about 400 cases per 100,000). If we compare the cumulative incidence in the reception system with the resident population by geographical macro-areas, we observe a 12% higher cumulative incidence in the reception system (with respect to Italian resident population) in the North, and about a 90% lower incidence in the rest of Italy.

The highest incidence of confirmed cases in the receiving system was observed in the Autonomous Province of Bolzano (6666.67; 95% CI: 4670.70–8662.63), Piedmont (1040.96; 95% CI: 781.09–1300.82), Veneto (854.09; 95% CI: 576.28–1131.90), and Lombardy (691.06; 95% CI: 518.24–863.88) regions.

Confirmed cases were significantly more frequent in CAS (82.4%) versus 7.5% in SIPROIMI and 10% in other/unspecified facilities (*p*-value < 0.00001) (Table 5); no confirmed cases were observed in CARA and centres for unaccompanied foreign minors (SIPROIMI MSNA). The cumulative incidence was 408 per 100,000 (95% CI: 351–465) for CAS and 235 per 100,000 (95% CI: 126–342) for SIPROIMI participating in the survey.

The saturation index, which overall reported a mean value of 79% for the facilities participating in the survey, was significantly higher in the 68 facilities with confirmed cases (87.7%) versus 78.6% of the facilities with no confirmed cases reported (*p* < 0.0001). Focusing attention on the geographical distribution, statistical significance remained for facilities located in Southern and Central Italy, while no difference in the saturation index between facilities with and without confirmed cases was found for facilities located in Northern Italy, which overall reported the highest saturation index.

#### 3.2.2. Confirmed Cases Characteristics

Among the 239 confirmed cases, 90.8% were male and 9.2% were female (Table 6), reflecting the sex distribution of guests in the Italian reception system. More than 50% were in the 25–39 age group while 33.5% of the cases presented symptomatology such as fever, fatigue, headache, dyspnoea, myalgia, arthralgia, and dry cough. Notably, body temperature of 37.5 °C or higher was reported in 67.5% of symptomatic cases.

Nearly twenty-six percent of confirmed cases (25.9%) were associated with hospitalisation (including only two in intensive care units), with an average length of stay of 20 days. All hospitalised cases were recorded in Northern Italy. Regarding symptoms, 56.5% of hospitalised cases had a temperature above 37.5 °C, 29% had headache, myalgia, or arthralgia, 24.2% had fatigue or dyspnoea, and 14.5% reported dry cough. The presence of symptoms was much less frequent among non-hospitalised cases. 

Fifteen (6.3%) of the confirmed cases reported comorbidities. The main comorbidities were cardiovascular, renal, and metabolic disorders (53.3% of all cases). Overall, 62 confirmed cases were hospitalised (177 non-hospitalised). Comorbidities were reported in seven cases out of the 62 hospitalised ones (11.3%) and in eight cases out of 177 non-hospitalised (4.5%) (*p* = 0.07) (Table 6). No deaths were observed.

All confirmed cases were reported to the local health authority, which ordered isolation within the centre in 25.5% of cases. Among positive cases isolated within centres, 31.1% were housed in single rooms with private facilities, 23% in a room shared with other positive cases, and 8.2% in a single room with private facilities.

## 4. Discussion

Italy was the first country in the world to put its entire territory into lockdown measures in early 2020. The first phase of the nationwide lockdown in Italy began on 9 March and ended on 4 May, when the measures were progressively weakened even though no movement among the Italian regions was yet allowed (Phase II). The third phase, characterized by the restart of working life and freedom of movement in the country, began on 15 June 2020. Due to the considerable number of migrants hosted in the Italian reception system, concern was expressed to protect the health of the hosted migrants and refugees in response to the pandemic. 

Local coordination and management of public health measures are carried out by the Departments of Prevention of local health authorities. Once informed of a suspected COVID-19 infection, the Departments test individuals and track contacts, also ensuring active surveillance of cases in fiduciary quarantine and isolation. 

The same public health measures apply to the reception system where, in a case of suspicion of COVID-19 positivity, the facility designated person has to inform the internal physician or directly the local health authority and implement all measures for temporary isolation until the Prevention Department performs the test and orders isolation. If the positive case does not require hospital treatment and safe isolation at the centre cannot be guaranteed, as for the general population, the local health authority has to consider, with the consent of the guest and in agreement with the prefecture and the managing body, to transfer the positive person to an appropriate external facility (COVID hotel or dedicated COVID facility in the reception system). Local health authorities are also in charge of identifying, testing, and quarantining the close contacts. Similarly, quarantined persons may be transferred to different dedicated facilities if safe preventive measures cannot be implemented at the facility itself. 

Our survey assessed the impact of COVID-19 pandemic in the reception system from the onset of the outbreak to the first and second phases of the national lockdown (March to 15 June 2020).

Despite the identification of outbreaks in some reception facilities, in our study, at national level, the incidence of COVID-19 in the reception system for migrants was globally substantially similar to that of the resident population in the same period, essentially showing no additional burden or risk of COVID-19 spread. If focusing on geographical macro-areas, the incidence of confirmed cases in reception centres, as being highest in the North and lowest in the South of Italy, reflected the North–South gradient of the Italian pandemic wave in the general resident population.

However, it is interesting to note that the North of Italy, which at the time of the survey was the macro-area with the highest circulation of the virus, registered 12% of incidence of cases in the reception system higher than that in the Italian resident population in the same macro-area; this suggests both a certain permeability of the facilities and some additional intra-centre risk factors. Conversely, in Central and Southern Italy, where the spread of the virus was initially limited, reception system facilities appeared to be much less affected than what expected. 

On this specific issue, our results seem to be in line with the data reported by Ceccarelli et al. who recorded in the same period, although with much smaller numbers (only one Italian reception centre with 300 migrants in the Lazio Region—Central Italy) the absence of cases of COVID-19 [19].

It is reasonable to hypothesize that in the North the highest circulation of the virus and the permeability of the system, e.g., due to operators who daily delivered services and goods in centres, played a certain role in increasing possible opportunities of contagions. If we add an intra-system risk factor, such as the high number of migrants hosted in the centres (in mean 11.8 in our study versus a mean of 2.3 in Italian households [20], with ratio 5:1) the incidence is even lower than expected.

Our results differ from those highlighted by Kondilis et al. which refer to migrants, refugees, and asylum seekers in reception and identification centres (RICs) and reception sites (RSs) of Greece. These data, that refer to the same period (first pandemic wave from 26 February to 30 June) emphasized that the risk of infection among migrants residing in IOM-managed RSs was almost 28 times higher than that of the general Greek population [21].

Regarding the characteristics of confirmed cases, comorbidities were found in 6.27% of cases versus 35.7% reported in the Italian resident population during the same period [22], a result that can be explained by the younger age of hosts and the overall good health status of migrants arriving in Italy. In contrast, a more pronounced average hospitalisation rate was observed (25.9% for COVID-19 cases in the host system versus 24% in March 2020 [23], 17.4% in April 2020 [24], 15.1% in May 2020 [22], and 7.2% in June 2020 [25] for the general Italian population). In this regard, the high rate of hospitalisation found in our study, even in guests without particularly severe symptoms and without comorbidities, may be related to factors other than clinical condition. 

More precisely, it cannot be ruled out that in the first phase of the pandemic the cause of the increased hospitalisation of migrants living in reception centres may lie in the precautionary attitudes of the staff of the facilities; in fact, the health professionals of the facilities may not know enough about the complete personal medical history of the migrant as well as they may not feel confident to manage possible clinical complications in the reception centre. 

Our study showed an overall positive response of the Italian reception system in limiting the spread of the pandemic within facilities, although some critical issues could be identified.

The North–South gradient of COVID-19 burden in the reception system, comparable to that of the Italian pandemic wave in the same lockdown period, suggests that some elements of permeability in the reception system must have however played a role, in particular in the North of Italy.

Moreover, if we look at facilities with clusters, we can affirm that overcrowding may represent a risk factor for the spread of the virus, highlighting the importance of physical distancing, reorganisation of activities, and adaptation of the internal areas of the reception facilities, in addition to an effective isolation of positive cases. However, according to our study, Italian reception centres have demonstrated a certain ability to delimit outbreaks, avoiding the occurrence of large epidemics within the centres.

In this regard, a critical situation was reported in Germany where, in the Ellwangen reception centre, COVID-19 cases increased from seven to 259 in one week, despite the facility being quarantined, and up to the end of April 2020, 68% of all 600 asylum seekers hosted had been infected, a situation that has raised concerns about effective compliance with preventive measures for COVID-19 [26]. 

Our study also showed that isolation of confirmed cases, ordered by local health authorities, was performed in the facility itself in a quarter of the cases, of which only 54.1% of the cases were in a single room with private facilities, a condition that, if not adequately managed (e.g., by isolating by cohort if there were no single rooms and private facilities), could pose a potential threat to the effective isolation of positive cases from healthy hosts.

The resilience proved by the Italian system of reception for migrants may be due to its structured governance and organisation. Italy has a well-structured and multi-level reception system that favours a widespread reception accommodation throughout the territory. Even the first assistance collective structures—where our analysis has highlighted some minor criticalities—are under the close guidance of the government and public institutions, supported by NGOs and other organisations. Unlike the Greek refugee camps where in some circumstances there were “severely substandard living conditions, poor sanitation, and lack of access to adequate or sometimes no health care” which hindered the implementation of preventive measures making them “virtually impossible” [21], the Italian government paid considerable attention to mitigating the risk of SARS-CoV-2 spread already in the first wave of the pandemic.

In this regard, the Italian Ministry of Interior on 18 March 2020 issued a circular letter to prefectures recommending facility managers to comply with COVID-19 preventive measures, while also suggesting the relocation of migrants to other facilities in case of overcrowding [27]. The Italian government had also timely introduced the execution of COVID-19 swab for all non-Schengen areas new arrivals and the preventive isolation of 14 days in dedicated facilities or in dedicated spaces within larger facilities to all new migrants entering the reception system. This measure would have certainly prevented the spread caused by possible infected new arrivals.

In addition, in August 2020, the INMP had published the “Interim operating procedures for the management of facilities with highly vulnerable persons at high risk of health and social exclusion during the COVID-19 outbreak” [28], a technical guidance document offering evidence-based recommendations to limit the spread of infection in both well-structured and unstructured care settings.

The document, based on international evidence published at the time including ECDC’s “Guidance on infection prevention and control of COVID-19 in migrant and refugee reception and detention centres” [10], addresses risk assessment of facilities, reorganisation of activities, adaptation of indoor areas, staff training, and guest education, providing instructions for early detection and management of suspected and positive cases in migrants from their first detection on Italian territory.

It is important to underline that our survey represents a huge national cohort study. It provides reliable national data because of the high coverage of facilities and the relative stability of the at-risk population during the lockdown phase. Nevertheless, a complete coverage of the population in the reception system and information about the male/female distribution of hosts in the system would have probably allowed a more accurate analysis of incidence. Moreover, a temporal analysis across the different phases of the national lockdown would have further enriched the results.

## 5. Conclusions

To the best of our knowledge, this is the first study in the world providing robust evidence both on COVID-19 cases detected in the reception system and on their related health outcomes. Although focusing on the first pandemic wave, the study allowed the testing of migrant reception system public health resilience over a pandemic period with a rapid increase of cases, highlighting in overall an epidemiological scenario in line with that observed in the rest of the country.

## Figures and Tables

**Table 1:** Geographical distribution of facilities participating in the survey (by region/AP and geographical macro-area).

Region/AP	Total *N* of Facilities	Facilities Participating in the Survey		
		*N*	% (Of the Regional Amount)	% (Of Facilities Participating in the Survey)
Piedmont	789	604	76.55	11.99
Aosta Valley	17	15	88.24	0.30
Lombardy	995	749	75.28	14.87
Autonomous Province of Bolzano	53	43	81.13	0.85
Autonomous Province of Trento	91	68	74.73	1.35
Veneto	476	343	72.06	6.81
Friuli Venezia Giulia	299	149	49.83	2.96
Liguria	328	217	66.16	4.31
Emilia-Romagna	990	855	86.36	16.97
Tuscany	475	275	57.89	5.46
Umbria	134	76	56.72	1.51
Marche	198	175	88.38	3.47
Lazio	552	459	83.15	9.11
Abruzzo	86	67	77.91	1.33
Molise	74	51	68.92	1.01
Campania	289	156	53.98	3.10
Apulia	264	204	77.27	4.05
Basilicata	122	99	81.15	1.97
Calabria	235	167	71.06	3.31
Sicily	295	212	71.86	4.21
Sardinia	75	54	72.00	1.07
**Macro-areas**				
Northern Italy	4038	3043	-	75.36
Central Italy	1359	985	-	72.48
Southern Italy and Islands	1440	1010	-	70.14
				*p* < 0.001
Total	6837	5038	73.69	100.00

AP: Autonomous Province.

**Table 2:** Reception centres characteristics.

Characteristics	*N* (%)
Participating facilities	5038 (73.7)
Geographical distribution	
Northern Italy	3043 (60.4)
Central Italy	985 (19.6)
South and Islands	1010 (20.1)
Typology	
First reception	
CAS	3750 (74.4)
CARA	4 (0.1)
Second reception	
SIPROIMI	1210 (24)
SIPROIMI MSNA	33 (0.7)
Others/unspecified	41 (0.8)
Accommodation	
Shared room	3450 (68.5)
Single room	507 (10.1)
Mixed	1081(21.5)
Average n. of guests (by centre)	11.8
First reception	
CAS	15.9
CARA	418.5
Second reception	
SIPROIMI	6.3
SIPROIMI MSNA	13.8
Others/unspecified	37.8
Facilities saturation index (%) (by geographical macro-area)	
Northern Italy	86.4
Central Italy	84.3
Southern Italy and Islands	66.3
	*p* < 0.001

CAS: Extraordinary Reception Centres; CARA: Reception Centres for Asylum Seekers; SIPROIMI: Protection System for Beneficiaries of International Protection and for Unaccompanied Foreign Minors; SIPROIMI MSNA: SIPROIMI facilities specifically dedicated to Unaccompanied Foreign Minors.

**Table 3:** Hosts characteristics.

Characteristics	*N* (%)
*N*	59,648
Geographical distribution	
Northern Italy	30,471 (51.1)
Central Italy	11,833 (19.8)
Southern Italy and Islands	17,344 (29.1)
Most represented nationalities	
Nigeria	16,207 (27.2)
Pakistan	7040 (11.8)
Gambia	4669 (7.8)
Bangladesh	3715 (6.2)
Senegal	3512 (5.9)

**Table 4:** Cumulative incidence of confirmed cases in the reception system and in the Italian resident population.

	Reception System		Italian Resident Population	
	Cumulative Incidence per 100,000	95% CI	Cumulative Incidence per 100,000	95% CI
Northern Italy	774.51	676.08–872.94	690.98	687.89–694.07
Central Italy	16.90	0.00–40.32	222.03	219.35–224.72
Southern Italy and Islands	5.77	0.00–17.07	95.14	93.80–96.49
Italy	400.68	349.99–451.38	396.21	394.61–397.80

CI: confidence interval.

**Table 5:** Distribution of confirmed cases by type of facility.

Type of Facility	Confirmed Cases	Total Hosts
CAS	197	48,294
CARA	0	1674
SIPROIMI	18	7676
SIPROIMI MSNA	0	454
Others/Unspecified	24	1550
Total	239	59,648

CAS: Extraordinary Reception centres; CARA: Reception Centres for Asylum Seekers; SIPROIMI: Protection System for Beneficiaries of International Protection and for Unaccompanied Foreign Minors. SIPROIMI MSNA: SIPROIMI facilities specifically dedicated to Unaccompanied Foreign Minors.

**Table 6:** COVID-19 confirmed cases characteristics.

Characteristics	*N* (%)
*N*	239 (0.4)
Sex	
Male	217 (90.8)
Female	22 (9.2)
Class Age (years)	
0–4	4 (1.7)
10–14	0
15–19	2 (0.8)
20–24	62 (25.9)
25–29	77 (32.2)
30–34	54 (22.6)
35–39	20 (8.4)
40–44	7 (2.9)
45–49	8 (3.4)
50–54	4 (1.7)
60–64	1 (0.4)
Clinical appearance	
Symptomatic	80 (33.5)
Asymptomatic	159 (66.5)
Comorbidities	
Yes	15 (6.3)
No	224 (93.7)
Hospitalised cases	62 (25.9)
With comorbidities	7 (11.3)
Without comorbidities	55 (88.7)
Non-hospitalised cases	177 (74.1)
With comorbidities	8 (4.5)
Without comorbidities	169 (95.4)

## Data Availability

All data relevant to the study are included in the article.
